# Ibrutinib, a Bruton’s tyrosine kinase inhibitor, exhibits antitumoral activity and induces autophagy in glioblastoma

**DOI:** 10.1186/s13046-017-0549-6

**Published:** 2017-07-17

**Authors:** Jin Wang, Xiaoyang Liu, Yongzhi Hong, Songtao Wang, Pin Chen, Aihua Gu, Xiaoyuan Guo, Peng Zhao

**Affiliations:** 1 Department of Neurosurgery, The First Affiliated Hospital, Nanjing Medical University, Nanjing, 210000 China; 2 Department of Intensive Care Unit, Shanghai General Hospital, Shanghai Jiao Tong University, School of Medicine, Shanghai, 201620 China; 3 Department of neurosurgery, Northern Jiangsu People’s Hospital, Yangzhou, 211406 China; 4 State Key Laboratory of Reproductive Medicine, Institute of Toxicology, Nanjing Medical University, Nanjing, 210000 China; 5 Key Laboratory of Modern Toxicology of Ministry of Education, School of Public Health, Nanjing Medical University, Nanjing, 210000 China; 6 Department of Neurosurgery, The Affiliated Zhong Da Hospital of Southeast University, Nanjing, 210009 China; 7 Department of Neurosurgery, Shengze Hospital of Jiangsu Province, Suzhou, 215228 China

**Keywords:** Anti-tumor, Autophagy, Glioblastoma, Ibrutinib

## Abstract

**Background:**

Glioblastoma (GBM) is the most common and aggressive primary brain tumor in adults. Ibrutinib, a Bruton’s tyrosine kinase (BTK) inhibitor, is a novel anticancer drug used for treating several types of cancers. In this study, we aimed to determine the role of ibrutinib on GBM.

**Methods:**

Cell proliferation was determined by using cell viability, colony formation, and 5-ethynyl-2′-deoxyuridine (EdU) assays. Cell cycle and cell apoptosis were analyzed by flow cytometry. Cell migratory ability was evaluated by wound healing assays and trans-well migration assays. ATG7 expression was knocked-down by transfection with *Atg7*-specific small interfering RNA. Overexpression of active Akt protein was achieved by transfecting the cells with a plasmid expressing constitutively active Akt (CA-Akt). Transmission electron microscopy was performed to examine the formation of autophagosomes in cells. Immunofluorescence and western blot analyses were used to analyze protein expression. Tumor xenografts in nude mice and immunohistochemistry were performed to evaluate the effect of ibrutinib on tumor growth in vivo.

**Results:**

Ibrutinib inhibited cellular proliferation and migration, and induced apoptosis and autophagy in LN229 and U87 cells. Overexpression of the active Akt protein decreased ibrutinib-induced autophagy, while inhibiting Akt by LY294002 treatment enhanced ibrutinib-induced autophagy. Specific inhibition of autophagy by 3-methyladenine (3MA) or *Atg7* targeting with small interfering RNA (si-Atg7) enhanced the anti-GBM effect of ibrutinib in vitro and in vivo.

**Conclusions:**

Our results indicate that ibrutinib exerts a profound antitumor effect and induces autophagy through Akt/mTOR signaling pathway in GBM cells. Autophagy inhibition promotes the antitumor activity of ibrutinib in GBM. Our findings provide important insights into the action of an anticancer agent combining with autophagy inhibitor for malignant glioma.

**Electronic supplementary material:**

The online version of this article (doi:10.1186/s13046-017-0549-6) contains supplementary material, which is available to authorized users.

## Background

Glioblastoma (GBM) is the most common and aggressive primary brain tumor in adults [1]. Despite considerable advances in the multimodal treatment of tumors, involving surgery followed by radio- and chemotherapy, only a minimal improvement in prognosis has been noted, with a median survival of less than 1 year [2]. The factors responsible for the limited efficacy of the current treatments include the highly invasive nature of GBMs, rendering them intractable to complete surgical resection, and resistance to conventional radiotherapy and chemotherapies [3, 4]. The development of novel drugs or overcoming the chemoresistance may therefore comprise a new line of research into the treatment of GBM.

Bruton’s tyrosine kinase (BTK) is a member of 11 tyrosine kinases, including the TEC family kinases, epidermal growth factor receptor (EGFR, ErbB1), ErbB2, ErbB4, Janus kinase 3 (Jak3), and BLK, that carry a conserved cysteine residue adjacent to an ATP-binding site; this residue is critical for covalent inhibition of these enzymes by tyrosine kinase inhibitors [5, 6]. Ibrutinib, formerly known as PCI-32765, selectively and irreversibly inhibits BTK, and is administered once-daily to prevent B-cell differentiation, proliferation, and survival [7]. Ibrutinib exerts a potent anti-cancer effect by inhibiting BCR signaling and down-regulating NF-кB signaling, rapidly reducing tumor growth by inhibiting tumor proliferation and increasing apoptosis [8–10]. Recently, ibrutinib has also been used as a novel anticancer drug for several other types of cancers, such as human ovarian, breast, and lung cancer, and also gastric carcinoma, and glioma [11–14]. Ibrutinib may also function as a novel small molecule inhibitor in GBM patients.

Autophagy (macroautophagy) is a “self-eating” process that enables the cell to engulf parts of its cytoplasm, organelles, and/or membrane through the formation of double-membrane vesicles (autophagosomes), and eventually targeting them to the lysosomes; this process is important for cell homeostasis, development, and/or nutrient recycling [15]. Under cellular stress conditions, such as nutrient deprivation, hypoxia, oxidative stress, DNA damage, etc., autophagy provides energy for the maintenance of essential cellular metabolism and enables cellular survival [16]. By promoting the survival of tumor cells under unfavorable conditions, autophagy may be involved in an alternative mechanism of drug resistance during cancer therapy. Recent extensive evidence indicates that autophagy is enhanced as a cytoprotective mechanism when cancer cells are subjected to unfavorable conditions, such as nutrient deficiency or treatment with chemotherapeutic drugs, aiding cancer cell survival. Temozolomide (TMZ) is widely used for treating primary and recurrent high-grade gliomas. Recent studies have shown that TMZ treatment can induce autophagy, which contributes to therapy resistance in glioma, and this has received considerable attention [17]; autophagy may also contribute to GBM resistance to anticancer therapies.

Autophagy is regulated by the main autophagy repressor, mammalian target of rapamycin (mTOR) complex 1 (mTORC1) [18]. It is inhibited by the intracellular energy sensor AMP-activated protein kinase (AMPK) [19]. Signaling pathways downstream of BTK, such as the PI3K/Akt pathway, are involved in the regulation of autophagy, indicating a potential link between ibrutinib and autophagy. The question of whether autophagy plays a role in cell death or constitutes a survival mechanism in GBM has not been investigated in detail.

In the current study, we investigated whether the modulation of autophagy may be used as an adjuvant modality to improve the effects of chemotherapy during GBM treatment. We performed a detailed analysis of the effect of ibrutinib on GBM cells. We demonstrated that ibrutinib exerts an antitumor effect and induces autophagy by targeting the Akt/mTOR signaling pathways in GBM. In addition, inhibiting macroautophagosome formation enhanced the GBM antitumor activity of ibrutinib. These findings provide important insights that may aid in the development of novel strategies to enhance the response of cancer cells to ibrutinib by exploiting the role of autophagy in GBM therapy.

## Methods

### Cell culture and chemicals

Human glioblastoma cell lines LN229, U87, T98, and U251 were purchased from the American Type Culture Collection (ATCC, Shanghai, China). All cells were routinely maintained in Dulbecco’s modified Eagle’s medium (DMEM) supplemented with fetal bovine serum (FBS, 10%; Gibco BRL, Grand Island, NY), nonessential amino acids (100 μM), sodium pyruvate (1 mM), streptomycin (100 μg/mL), and penicillin (100 U/mL, Gibco BRL) at 37 °C, in an atmosphere of 5% CO_2_. Ibrutinib and LY294002 were obtained from Selleck Chemicals (Houston, TX) and were dissolved in dimethyl sulfoxide (DMSO; Sigma, St. Louis, USA) at a concentration of 10 mM. The final concentration of DMSO in treatment did not exceed 0.1% (v/v). 3-Methyladenine (3MA) was purchased from Sigma-Aldrich (St. Louis, USA) and dissolved in phosphate-buffered saline (PBS, Gibco BRL) at a concentration of 100 mM. Before use, stock solutions were diluted to the required concentrations in culture medium.

### Cell viability assay

Cell viability was analyzed using a WST-8 Cell Counting Kit-8 (CCK-8, Beyotime, Jiangsu, China). U87, U251, and LN229 cells were suspended in DMEM medium containing 10% of FBS (3 × 10^3^ cells/100 μL) and were seeded in 96-well plates and treated with different concentrations of chemicals, as specified. At the indicated time points, the cells were stained with CCK-8 (10 μL/well) and the cultures were incubated at 37 °C for 90 min. The absorbance at 450 nm was measured using an immunoreader (Infinite M200; Tecan, Männedorf, Switzerland).

### Colony formation assay

Cells (200 cells per well) were counted and 1.0 × 10^4^ cells were seeded in 6 × 6 cm plates in DMEM supplemented with 10% of FBS. The cells were treated with the indicated agents for 10 day. Colonies were stained with 0.2% crystal violet solution (Beyotime) and counted after 10 day of incubation at 37 °C and 5% CO_2_. Clusters of cells containing over 50 cells were counted as a colony. For each clone, three independent plates were examined.

### 5-ethynyl-2′-deoxyuridine (EdU) proliferation assay

GBM cell proliferation was determined in vitro using the Cell-Light™ EdU DNA cell proliferation kit (Ribobio, Guangzhou, China) according to the manufacturer’s instructions.

### Small interfering RNA (siRNA) and plasmid construction

Cells were seeded (2 × 10^5^ cells/well) in 6-well plates. After a 24-h incubation, the cells were transfected with siRNA targeting *Atg7* (GenePharma, Shanghai, China), using Lipofectamine 2000 (Invitrogen, Carlsbad, CA). The sequences of interference were as follows: si-Atg7, 5′-CAGCCUGGCAUUUGAUAAATT-3′ (sense) and 5′-UUUAUCAAAUGCCAGGCUGTT-3′ (antisense); si-NC, 5′-UUCUCCGAACGUGUCACGUTT-3′ and 5′-ACGUGACACGUUCGGAGAATT-3′. Constitutively-active Akt (CA-Akt) and dominant-negative Akt (DN-Akt) plasmids were constructed by Sunbio (Shanghai, China).

### Cell migration assay

Cell migration was assessed in wound healing assays and trans-well migration assays. For the wound healing assays, 5 × 10^5^ cells/well were plated in 6-well dishes, and incubated with various concentrations of ibrutinib at 37 °C overnight. A cell-free gap was generated by scratching dishes with a 10-μL pipette tip. For trans-well migration assay, the cells were re-suspended in a serum-free DMEM medium (3× 10^5^ cells/200 μL) with ibrutinib and then seeded into the upper chamber, over 8-μm pore polycarbonate filters (Millipore, Massachusetts, USA). A serum-containing DMEM medium (600 μL) was placed in the lower chamber. After 24 h of incubation, the cells that migrated to the bottom of the membrane were attached and fixed, and stained with 0.2% crystal violet solution.

### Western blot analysis

Drug- or vehicle-treated cells, or mouse tissue samples were lysed in a lysis buffer containing 20 mM Tris (pH 7.5), 150 mM NaCl, 1% Triton X-100, 2.5 mM sodium pyrophosphate, 1 mM EDTA, 1% Na_3_VO_4_, 0.5 μg/mL of leupeptin, and 1 mM phenylmethanesulfonyl fluoride (PMSF; Beyotime). Protein concentrations were measured using the Bio-Rad protein assay (Bio-Rad Laboratories, Hercules, CA). The samples were then scraped and transferred into microfuge tubes, centrifuged at 12,000 rpm for 15 min, and heated in an SDS-PAGE protein loading buffer (Beyotime) at 95 °C for 10 min. Equal amounts of protein were separated on 10 or 15% SDS-PAGE gels (Beyotime). After the electrophoresis, the separated proteins were transferred to a PVDF membrane (Beyotime); the membranes were then blocked in 5% of nonfat milk for 60 min. Next, the membranes were incubated overnight at 4 °C with the following primary antibodies raised against: phospho-GSK3β (Ser9) (#5558), phospho-BTK (#5082P), BTK(#8547), phospho-Akt(#9271), Akt (#9272), LC-3A/B (#12741), Atg (#8558), cyclin D1 (#2922), p-Rb (#3590), p-mTOR(#5536), mTOR(#2972), p-ULK1(#12753), ULK1(#8054), p-p70S6K (#9208), p70S6K(#14130), cleaved caspase 3 (#9661), cleaved caspase 9 (#9502), and Bcl-xL (#2764), from Cell Signaling technology (Danfoss, USA); GAPDH (AG019), from Beyotime; or E2F1 (ab179445), from Abcam (Cambridge, UK). Following a 1-h incubation with horseradish peroxidase (HRP)-labeled secondary antibodies, the blots were developed using a western blot chemiluminescence reagent system (Perkin-Elmer, NEL103001EA, Waltham, USA). Three replicates were performed for each experiment.

### Transmission electron microscopy (TEM)

To assess cell morphology by electron microscope, the treated cells were fixed in 3% glutaraldehyde, post-fixed in 1% osmium tetroxide solution, dehydrated with acetone, and embedded in Epon resin (Agar Scientific, Stansted, UK). Ultrathin sections were prepared with an Ultracut microtome (Leica, Oskar-Barnack, Germany) and then stained with 4% uranyl acetate and lead citrate. The sections were examined using a JEM-100cxII electron microscope (JEM-1010, JEOL, Tokyo akishima, Japan).

### Immunocytochemistry

GBM cells were fixed and permeabilized in 0.2% Triton X-100 (). After washing with xx, the cells were blocked with 5% BSA, incubated with specific antibodies against LC-3A/B (1:50, Neomarkers, Fremont, CA), overnight at room temperature, followed by an incubation with Cy3-labeled goat anti-rabbit antibodies (1:200, Beyotime). Finally, the coverslips were removed and mounted onto glass slides in Vectashield mounting medium containing DAPI (Vecta Laboratories, Burlingame, CA). Images were acquired with a laser scanning microscope (Infinite M200 Pro, Tecan); LSM510 software was used to capture the images (Zeiss, Aobokeheng, Germany).

### Flow cytometry analysis

GBM cells treated with drugs or DMSO were trypsinized, suspended in ice-cold PBS, and fixed in 70% ethanol at −20 °C. Cell cycle progression was evaluated using BD Cycletest Plus kit and BD FACS Calibur flow cytometer (BD, Franklin Lakes, NJ). After fixing, the cells were washed twice with PBS, stained in 250 μL of trypsin buffer for 15 min, and eventually added to 200 μL of trypsin inhibitor with RNase buffer. The samples were finally stained with 200 μL of PI solution and analyzed.

Cell apoptosis was analyzed using BD annexin V-fluorescein isothiocyanate (FITC)/PI apoptosis detection kit. Harvested cells were washed with cold PBS, resuspended in 50 μL of annexin binding buffer, stained with 5 μL of annexin V-FITC and 5 μL of PI solution for 15 min at room temperature in the dark, and then diluted in 400 μL of 1× binding buffer.

### Tumorigenicity in nude mice

BALB/C nude mice (4–5 week-old) were provided by the animal center at the Cancer Institute at the Model Animal Research Center of Nanjing University (Nanjing, China) and randomly divided into four groups (control group, Ib group, 3MA group, and Ib + 3MA group). U87 cells (2 × 10^6^) in 100 μL of serum-free DMEM were injected into the right flank of mice. Tumor volume was assessed every 3 days. Mice were injected intraperitoneally (i.p.) every other day, starting on day 3, with PBS alone (control), ibrutinib (6 mg/kg/d), 3MA (30 mg/kg/d), or ibrutinib (6 mg/kg/d) and 3MA (30 mg/kg/d). The tumor and body weights were determined. Tumor volume was calculated by the following formula: (short diameter)^2^ × (long diameter)/2. Mice were humanely sacrificed on day 22. For immunohistochemical analysis, samples from each group of mice were stained with H&E and a primary antibody (rabbit anti-LC3A/B or rabbit anti-Ki67, both at 1:200 dilution).

### Statistical analysis

Data are expressed as the mean ± standard deviation (SD) from at least three independent experiments. Student’s *t* test was performed to assess statistical significance using GraphPad Prism (GraphPad, San Diego, CA). A value of *p* < 0.05 was regarded as statistically significant.

## Results

### Ibrutinib inhibits proliferation of GBM cells

**Fig. 1:**
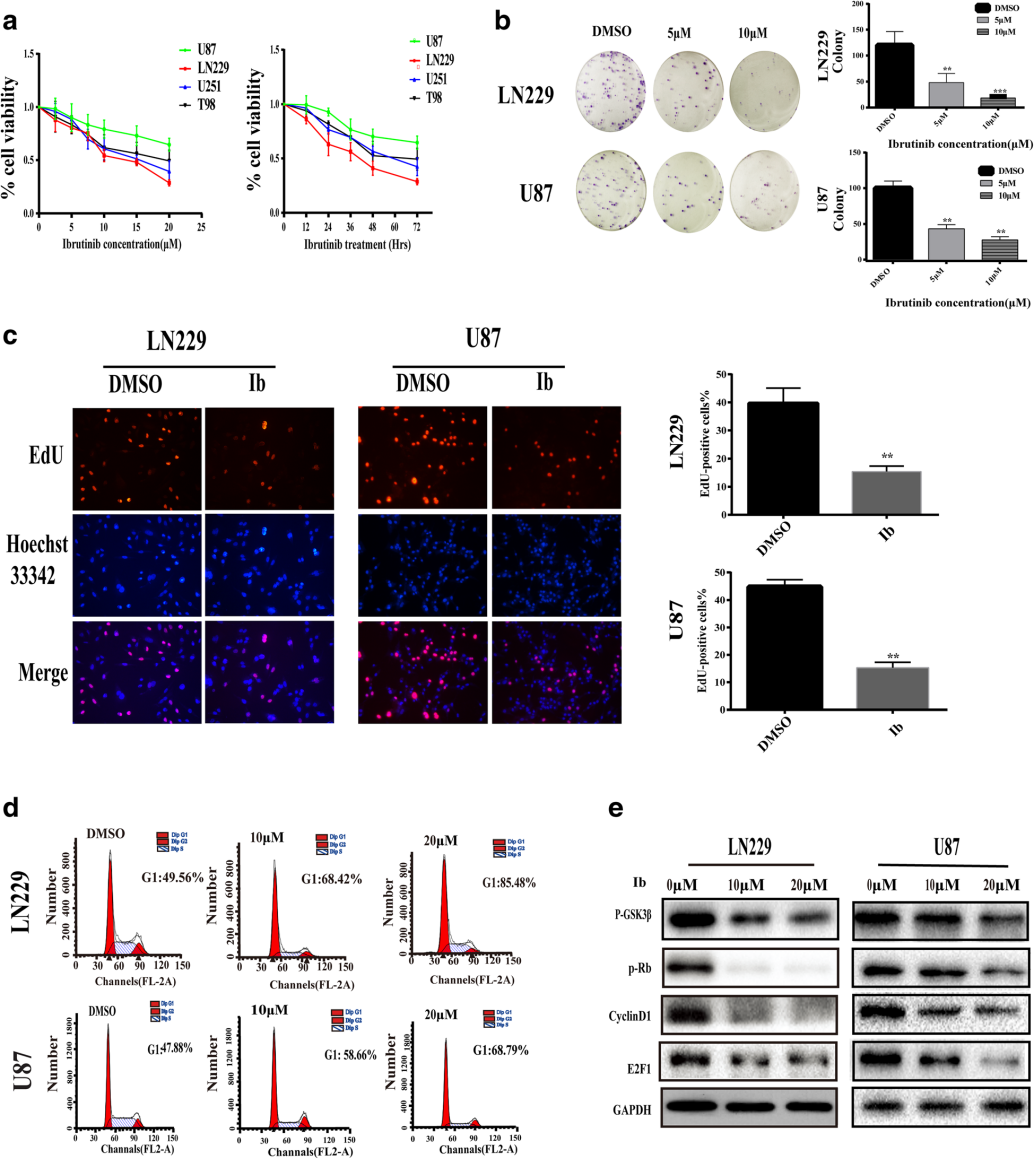
Ibrutinib inhibits the proliferation of GBM cells. (**a**) The concentration- (*left*) and time- (*right*) dependent effect of ibrutinib (Ib) on GBM cell viability was assessed using CCK8 assay (the data are presented as the mean ± SEM, *n* = 4 biological replicates). (**b**) U87 and LN229 cells were treated with different concentrations of ibrutinib (0, 5, or 10 μM) for 10 day. The formation of cell colonies was then evaluated (the data are presented as the mean ± SEM, *n* = 3 biological replicates); *p < 0.05 and **p < 0.01 compared with the control group (DMSO). (**c**) The proliferation capacity of GBM cells treated with ibrutinib (10 μM) for 24 h, as determined by EdU assay. Proliferating cells are stained red and cell nuclei are stained with Hoechst 33,342 (*blue*); **p < 0.01 compared with the control group (DMSO). (**d**) The effect of ibrutinib on proliferation of LN229 and U87 cells, as examined by flow cytometry. (**e**) Western blot analyses of GSK3β, p-Rb, cyclinD1, E2F1, and GAPDH protein levels in LN229 and U87 cells after treatment with different concentrations of ibrutinib for 24 h

Ibrutinib is a highly effective BTK inhibitor used for the treatment of B-cell malignancies. We noted that the expression of BTK in GBM tissues deposited in the Cancer Genome Atlas (TCGA) and Gene Expression Omnibus (GSE7696, GSE16011) is up-regulated (Additional file 1: Figure S1). To determine the effect of ibrutinib on the viability of human glioma cells, U87, LN229, T98, and U251 cells treated with different concentrations of ibrutinib for 72 h were analyzed by CCK8 assay (Fig. 1a). The results indicated that ibrutinib decreased GBM cell viability in a dose-dependent manner. Furthermore, cell viability was also reduced with increasing treatment time (Fig. 1a). To evaluate the long-term effect of ibrutinib on cell survival, colony formation assay was performed. A significant reduction in the number of colonies was observed when GBM cells were treated with different concentrations of ibrutinib (0, 5, or 10 μM) (Fig. 1b). The EdU incorporation assay suggested that ibrutinib attenuated cell proliferation in both LN229 and U87 cells in a dose-dependent manner (Fig. 1c). As revealed by flow cytometry evaluation, GBM cells were arrested in the G0/G1 phase (Fig. 1d). Cell cycle progression is promoted by cyclin-dependent kinases (CDK), cyclins, and inhibited by CDK inhibitors, including cyclin D1, E2F1, and Rb phosphorylation. It is known that the expression of cyclin D1 is mediated by GSK-3β [20]. As determined by western blotting with specific antibodies, exposure to ibrutinib led to a decrease in cyclin D1, E2F1, and phosphorylated Rb levels, and also a decrease in p-GSK3β levels (Fig. 1e); this suggested that inhibition of cyclin D1 and E2F1 expression, and of Rb and GSK3β phosphorylation, might play a role in ibrutinib-induced G1 arrest in GBM cells.

### Ibrutinib suppresses migration and induces apoptosis of GBM cells

**Fig. 2:**
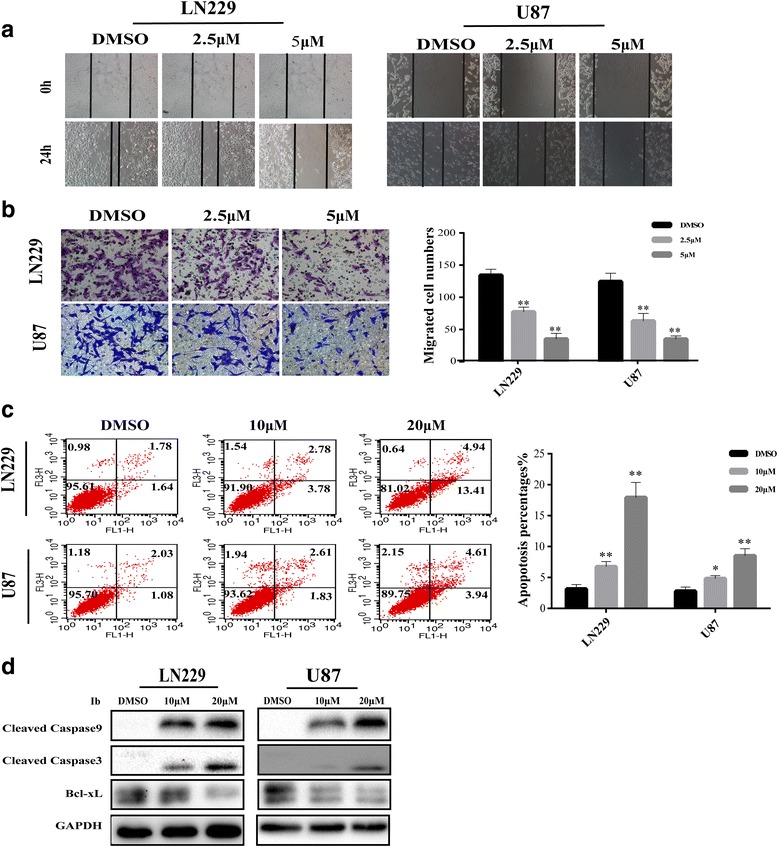
Ibrutinib suppresses cell migration and induces apoptosis in GBM cells. (**a**) The migratory ability of LN229 and U87 cells was evaluated in a wound healing assay with cells treated with various concentrations of ibrutinib for 24 h. (**b**) The results of trans-well assay with LN229 and U87 cells treated with different concentrations of ibrutinib for 24 h. Statistical analyses of the migrated cells are shown on the right; **p < 0.01. (**c**) The percentage of apoptotic cells in LN229 and U87 cell population treated with increasing concentrations of ibrutinib, as detected by flow cytometry with annexin V-PI staining. Data are shown as the mean ± SD and are from three independent experiments; *p < 0.05, **p < 0.01. (**d**) The expression of apoptosis-associated proteins cleaved caspase 9, cleaved caspase 3, and Bcl-xL were detected by western blotting following the treatment of cells with increasing concentrations of ibrutinib for 48 h

GBM cells are not only characterized by infinite proliferation ability but also by high migration and anti-apoptosis ability. Wound healing and trans-well migration assays were used to examine the possible effects of ibrutinib on cell migration. As shown in Fig. 2a, the scratch area was significantly larger in untreated GBM cells (LN229 and U87) than in ibrutinib-treated cells after 24 h of ibrutinb treatment. The trans-well migration assays revealed that the number of LN229 or U87 cells in the lower chamber was significantly reduced by ibrutinb treatment in a dose-dependent manner (Fig. 2b). Flow cytometry was used to determine the effect of ibrutinib on the apoptosis of human GBM cells; the population of apoptotic LN229 and U87 cells increased upon ibrutinib treatment, indicating that ibrutinib potently induced apoptosis in GBM cells (Fig. 2c). Activation of caspase 9 activates caspase 3, which initiates apoptosis, and we therefore examined the involvement of caspases in ibrutinib-induced apoptosis. As evidenced by western blotting analysis, the cleavage of caspase 3 and 9 increased in a dose-dependent manner upon ibrutinib treatment (Fig. 2d). In addition, the levels of anti-apoptotic Bcl-xL protein significantly decreased in ibrutinib-treated cells (Fig. 2d).

### Ibrutinib induces autophagy in GBM cells

**Fig. 3:**
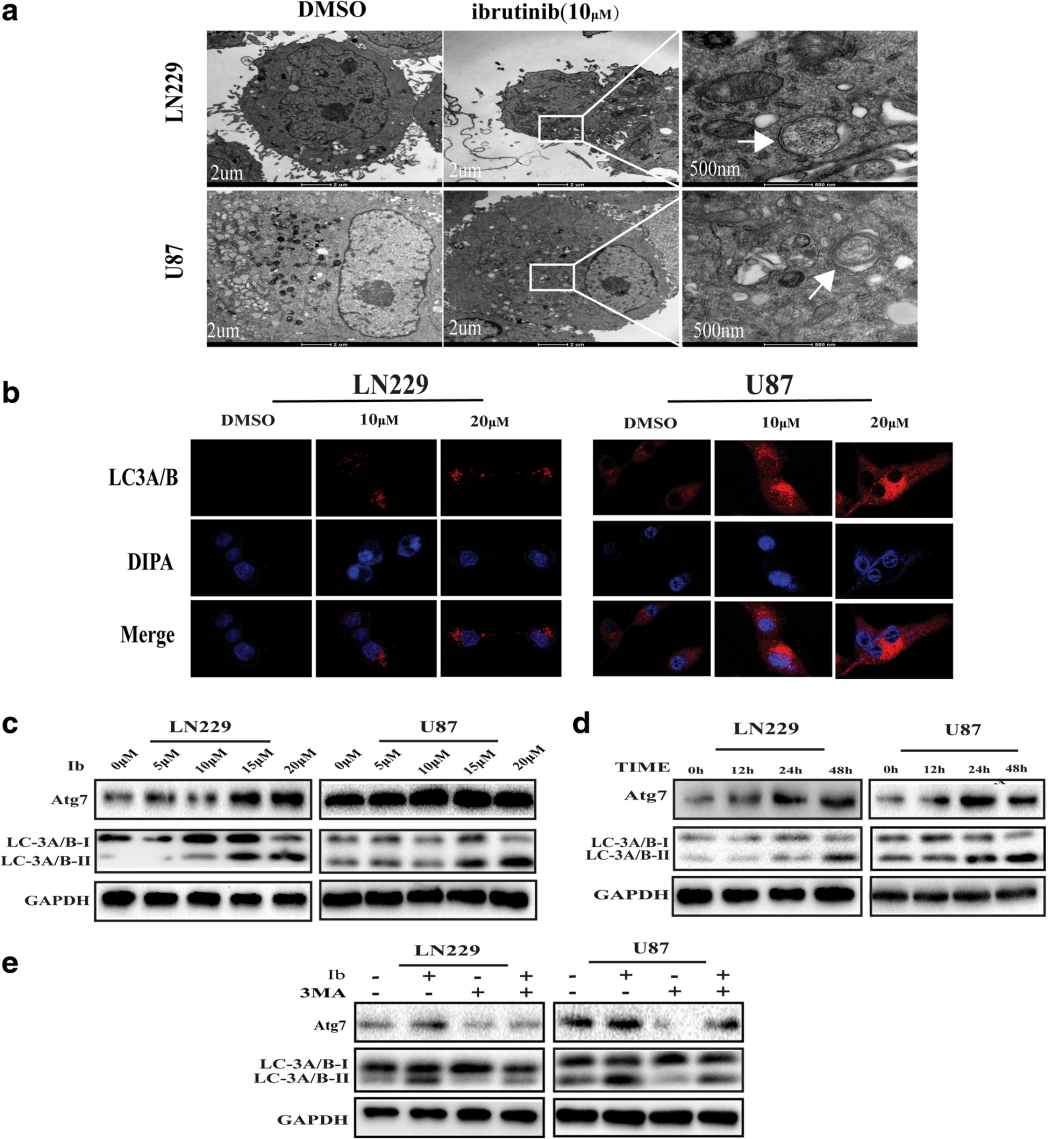
Ibrutinib induces autophagy in GBM cells. (**a**) TEM revealed autophagosome ultrastructures in the enlarged images (arrows) after a 24-h treatment with 10 μM ibrutinib. (**b**) Representative images of immunocytochemistry. Red fluorescence indicates the presence of LC-3 protein. (**c**, **d**) GBM cells were incubated with different concentrations of ibrutinib for 24 h (**c**) or with 10 μM ibrutinib for various times (**d**), and LC3A/B-II, Atg7, and GAPDH levels were assessed by immunoblotting. (**e**) LC3A/B and Atg7 levels examined by western blot analysis in LN229 and U87 cells after treatment with ibrutinib (10 μM) or DMSO, in the absence or presence of 3MA (2 nM)

We next investigated the occurrence of autophagy in ibrutinib-treated LN229 and U87 GBM cells. TEM is the gold standard for detecting autophagosome formation since the autophagosomes have characteristic double-membrane or multi-membrane structures. Double- or multi-membrane structures were indeed accumulating in LN229 and U87 cells treated with 10 μM ibrutinib, indicating the formation of autophagosomes (Fig. 3a). We next used specific LC3A/B antibody and confocal microscopy to examine the conversion of LC3A/B-I to LC3A/B-II. As shown in Fig. 3b, the intensity of punctate LC3A/B fluorescence increased in LN229 and U87 cells upon a 24-h treatment with 10–20 μM ibrutinib. Immunoblotting of lysates of ibrutinib-treated GBM cells revealed a significant increase of processed LC3A/B-II and Atg7 proteins in a dose- and time-dependent manner; these proteins are critical components in regulating the formation of autophagosomes [21] (Fig. 3c and d). Consistent with these observations, incubation with 3MA, an autophagosome formation inhibitor. A combined ibrutinib and 3MA treatment decreased the LC3A/B-II levels (Fig. 3e), indicating that ibrutinib induced autophagy in GBM cells.

### Ibrutinib induces autophagy in GBM cells by targeting the Akt/mTOR pathway

**Fig. 4:**
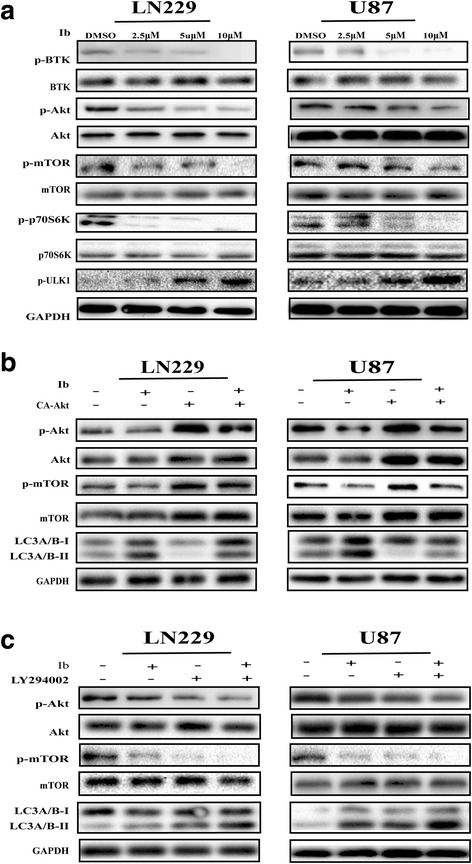
Ibrutinib induces autophagy in GBM cells by targeting the Akt/mTOR pathway. (**a**) Western blot analysis of p-BTK,BTK, p-Akt, Akt, p-mTOR, mTOR, p-p70S6K, p70S6K, p-ULK1, ULK1 and GAPDH levels in LN229 and U87 cells following a 24-h treatment with increasing concentrations of ibrutinib. (**b**) After the cells were treated with ibrutinib for 24 h in the presence or absence of pcDNA3-CA-Akt plasmid, the cells were treated with ibrutinib (10 μM) for 24 h, and p-Akt, Akt, p-mTOR, mTOR, LC3A/B, and GAPDH levels were evaluated by western blotting. (**c**) p-Akt, Akt, p-mTOR, mTOR, LC3A/B, and GAPDH levels determined by western blotting in LN229 and U87 cells pretreated with LY294002 and then treated with ibrutinib for 24 h

The mammalian target of the Akt/mTOR pathway is a key regulator of autophagy [22]. Previous studies have shown that ibrutinib inhibits GBM oncogenicity through BTK/Akt/mTOR pathway [14]. We observed that the levels of phosphorylated Akt, mTOR, and p70 ribosomal protein S6 kinase (p70S6K) were significantly down-regulated and the levels of phosphorylated UNC-51-like kinase 1 (ULK1) were increased in ibrutinib-treated cells (Fig. 4a). Next, we investigated whether the Akt/mTOR pathway is involved in ibrutinib-induced autophagy in LN229 and U87 cells. As shown in Fig. 4b, overexpression of constitutively-active Akt by transfecting the cells with pcDNA3-CA-Akt plasmid decreased LC3A/B-II expression, and this effect was enhanced by ibrutinib treatment. When the LN229 and U87 cells were pretreated with LY294002, an inhibitor of the PI3K/Akt/mTOR signaling pathway, the levels of ibrutinib-induced autophagy protein IC3A/B-II were markedly increased in LN229 and U87 cells (Fig. 4c). These results indicated that the Akt/mTOR signaling pathway is a critical mediator regulating the ibrutinib-induced autophagy.

### Blocking autophagy enhances ibrutinib-induced cell death

**Fig. 5:**
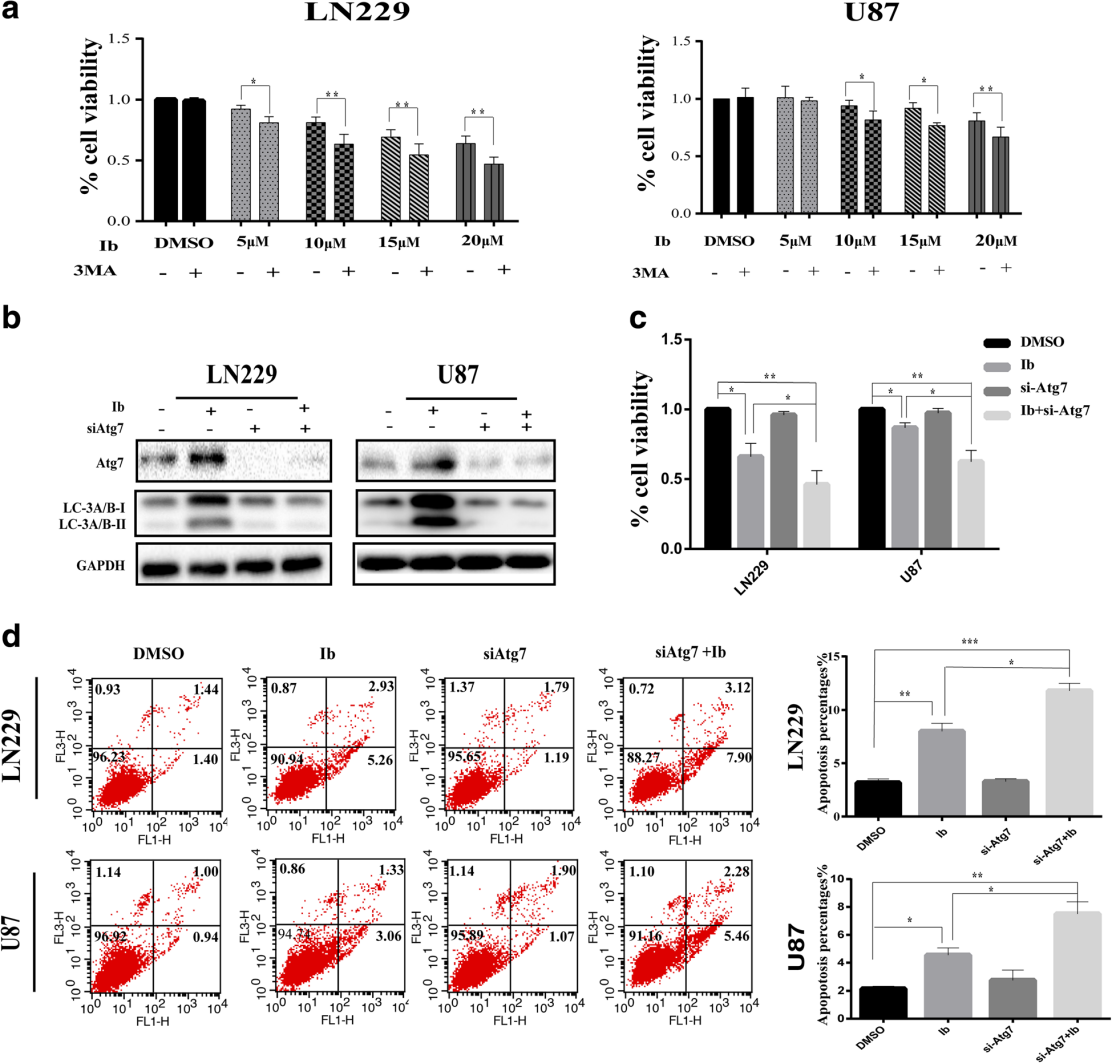
Inhibition of autophagy enhances the antitumor efficacy of ibrutinib in LN229 and U87 cells. (**a**) GBM cells were incubated with or without the autophagy inhibitor 3MA (2 mM) for 1 h, and then treated with various concentrations of ibrutinib for 24 h. Cell viability was evaluated by CCK8 assay. Data are presented as the mean ± SEM (*n* = 3); *p < 0.05, **p < 0.01, compared with the control (no treatment). (**b**) The cells were treated with ibrutinib in the presence or absence of small interfering RNA (si-Atg7). Atg7 and LC3A/B levels were then determined by western blotting. (**c**) LN229 and U87 cells were treated with ibrutinib with or without a prior 24-h transfection with si-Atg7. CCK8 assay was then used to analyze cell viability; *p < 0.05, **p < 0.01. (**d**) Flow cytometry analysis of cell death. The data are shown as the mean ± SEM. The experiments were performed in triplicate; *p < 0.05, **p < 0.01, ***p < 0.01

Many studies have demonstrated that autophagy may serve as a protective cell response preventing tumor cells from therapy-induced cell death [23–25]. To investigate whether the autophagy-inducing activity of ibrutinib contributes to its antitumor activity, CCK8 assay was used to compare cell viability after treatment with ibrutinib alone and in combination with autophagy inhibitor, 3MA. The results revealed that ibrutinib/3MA co-treatment potentiated the cytotoxic effects of ibrutinib (Fig. 5a). To further explore the relationship between the autophagy and ibrutinib-induced cell death, we silenced the expression of *Atg7* in LN229 and U87 cells with a small interfering RNA. After transfection with si-*Atg7*, the Atg7 and LC3A/B-I/II protein levels were significantly down-regulated in ibrutinib-treated cells (Fig. 5b). Moreover, transfection with si-*Atg7* enhanced ibrutinib-induced decline in cell viability (Fig. 5c). Further, knock-down of *Atg7* significantly enhanced ibrutinib-induced apoptosis in GBM cells (Fig. 5d). Collectively, these results suggested that the inhibition of autophagy promotes the cytotoxic effect of ibrutinib in GBM cells.

### Ibrutinib induces autophagy and exerts antitumor effect in U87 xenograft model

**Fig. 6:**
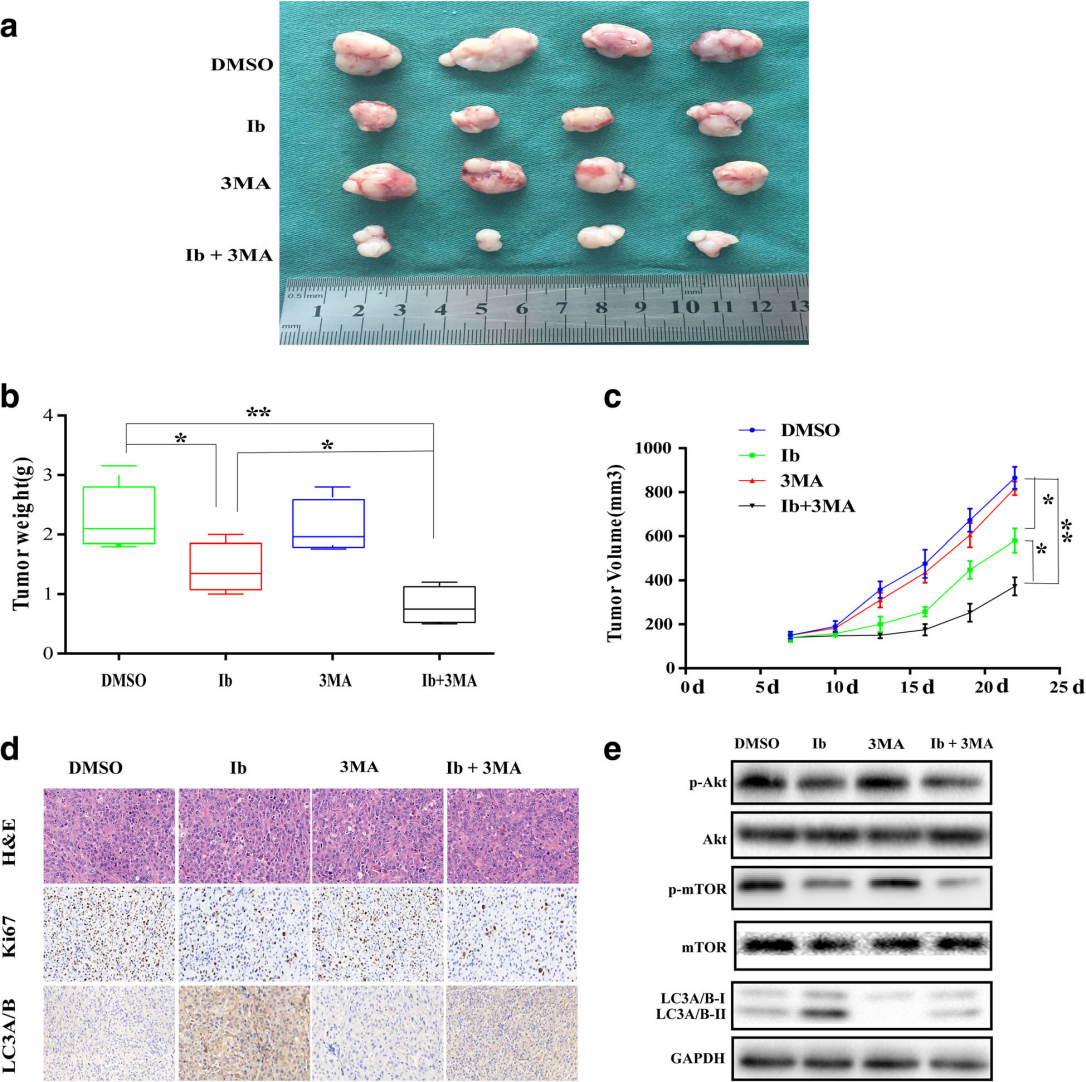
3MA treatment enhances antitumor efficacy of ibrutinib in U87 xenograft model. Mice were sacrificed 22 days after the indicated treatments. The tumors were isolated (**a**), and tumor weight (**c**) and volume (**d**) were measured; *p < 0.05, **p < 0.01. (**e**) Analysis of tumors from each group by H&E staining and immunohistochemical detection of LC3A/B and Ki67. (**f**) Western blot analysis of p-Akt, p-mTOR,mTOR, LC3A/B, and GAPDH levels in isolated tumors

Next, the potential usage of ibrutinib in combination with 3MA was evaluated in vivo. Mice with implanted U87 cells were randomly assigned into four experimental groups (control group, ibrutinib group, 3MA group, and ibrutinib + 3MA group). As shown in Fig. 6a–c, anti-tumorigenic effect was observed in the ibrutinib group and ibrutinib + 3MA group. Moreover, ibrutinib in combination with 3MA appeared to be more effective than ibrutinib-only treatment, as evidenced by xenograft model. H&E staining did not reveal any differences in histology among the four groups (Fig. 6d). The immunohistochemical (IHC) examination of tissues from ibrutinib group and ibrutinib + 3MA group revealed that the ibrutinib/3MA combination decreased the number of Ki67-positive cells and LC3A/B levels to a greater extent than ibrutinib-only treatment; the inhibition of autophagy by 3MA did not lead to a greater decrease of Ki67-positive cells in 3MA group than in the control group (Fig. 6d). The data suggested that a combination treatment with 3MA promoted anti-tumorigenic effect of ibrutinib in vivo. To further elucidate the role of the Akt/mTOR pathway in ibrutinib-induced autophagy, we evaluated the expression of LC3A/B, p-Akt, and p-mTOR in the mice tumor by western blotting. As shown in Fig. 6e, p-Akt and p-mTOR levels were increased during ibrutinib treatment together with increasing LC3A/B-II expression. Taken together, these results confirmed that autophagy inhibition promotes antitumoral activity of ibrutinib in GBM.

## Discussion

The poor prognosis of GBM under current therapeutic regiments has necessitated the development of novel therapeutic agents. In the current study, we explored the potential anticancer effect of ibrutinib in GBM. Our results indicate that ibrutinib indeed has a pronounced anticancer potential in GBM. Furthermore, we also confirmed that ibrutinib induces autophagy by targeting Akt/mTOR pathway. Finally, we provided evidence that the blockage of autophagy can potentiate the effect of ibrutinib on GBM in vitro and in vivo.

Autophagy has generally been considered to facilitate cancer survival during growth factor withdrawal or under metabolic stress, e.g., gamma-radiation, exposure to toxic stimuli, and chemotherapy [26, 27]. This process plays an important role not only during different stages of tumorigenesis but also during disease, creating a tumorigenesis-promoting microenvironment [28]. In certain cellular settings, however, it was reported that autophagy might suppress tumorigenesis by inducing autophagic cell death [29, 30]. Thus, the current view of autophagy in tumorigenesis is as of a double-edged sword that can either act as a tumor suppressor or promoter; this issue is receiving increasing scientific attention. Recently, a number of cancer therapeutics in cancer indicated that autophagy can be activated and protect tumor cells when they are exposed to targeted therapies, e.g., Philadelphia chromosome-positive cells and imatinib mesylate [31], breast cancer and trastuzumab [32], prostate cancer and Src family kinase inhibitors [33], and prostate cancer and proteasome inhibitors [34]. Autophagy-related genes, ATGs, function at several discrete but continuous steps of autophagy. Upon the induction of autophagy, some LC3(*Atg8*)-I (LC3-I) synthesized in the cytoplasm is evenly converted to LC3-II, which is tightly bound to the autophagosomal membranes, forming ring-shaped structures in the cytosol. LC3 expression is positively correlated with GBM patient survival and performance status, whereas in patients with normal performance scores, low LC3 expression correlates with better survival [35]. The combination of TMZ, the most effective drug for GBM treatment, and autophagy inhibitors [e.g., chloroquine (CQ) and its analogs] has attracted attention in a rational development of therapeutic approaches, and is under clinical trials as GBM treatment [17]. These suggested that autophagy maybe be activated as a cellular response to GBM therapy. In the current study, we confirmed that autophagy is induced by ibrutinib, as determined by TEM and immunocytochemistry. Western blot analysis showed that ibrutinib increases LC3-II protein levels in a concentration- and time-dependent manner, in two independent GBM cancer cell lines. Hence, autophagy may indeed be activated as a cellular response to GBM therapy.

Autophagy is a complex process, fine-tuned by several environmental signals involved in nutrient signaling, growth factor status, energy sensing, hypoxia, oxidative and ER stress, and infection [36]. AMPK and mTOR signaling pathways have been revealed as the central checkpoints in the regulation of autophagy [37]. It has been reported that ibrutinib suppresses GBM tumorigenesis by inhibiting BTK and its downstream Akt/mTOR signaling [14]. Our results revealed that ibrutinib treatment inhibits BTK activation and phosphorylation of its downstream targets, including Akt, mTOR, and p70S6K. Accumulating evidence has highlighted the notion that the inhibition of Akt and its downstream targets mTOR and p70S6K contributes to the initiation of autophagy [38]. In the current study, overexpression of constitutively-active Akt markedly decreased ibrutinib-induced autophagy. In contrast, PI3K/Akt/mTOR signaling pathway inhibitor LY294002 enhanced ibrutinib-induced autophagy. The Akt/mTOR signaling pathway is therefore a critical mediator regulating ibrutinib-induced autophagy. Aberrant EGFR signaling, expression of EGFR vIII mutant interact with the PI3K/Akt/mTOR pathway were frequently observed in GBM patients, promoting survival and chemo-resistance [39]. Gao et al. [40] recently reported that ibrutinib selectively inhibits growth of mutated NSCLC cells, including T790M mutant and erlotinib-resistant H1975 cells, by inhibiting EGFR phosphorylation [40]. Hence, ibrutinib may also induce autophagy along the RTK-PI3K-Akt-mTOR axis. Autophagy induction by targeting the components of the PI3K-Akt-mTOR axis has been typically suggested to play a cytoprotective role in GBM﻿. Combination of bafilomycin A1 or monensin, which inhibits lysosomal protease activity, with PI-103 or Ku-0063794, mTOR kinase inhibitors, promoted GBM cell death by inducing apoptosis [41]. A combination of PI3K/mTOR/Akt inhibitors PI-103 and Akt-1/2 with the lysosomotrophic agent CQ enhanced cell death in GBM [42]. Additionally, a combination of a dual PI3K and mTOR inhibitor, NVPBEZ235, with CQ induced apoptosis of glioma cells [41]. Similarly, suppression of autophagy has been reported to synergize with Tyrosine Kinase Inhibitor (TKI), such as erlotinib [43] or imatinib [44], to increase the cytotoxic effect on GBM cells. In the current study, knocking-down *Atg7* significantly enhanced the ibrutinib-induced apoptosis of glioma cells in vitro. Moreover, we also confirmed that the blockage of autophagy by 3MA increased the anti-cancer effect of ibrutinib on GBM in vivo.

## Conclusions

Taken together, ibrutinib exerts a profound antitumor effect on GBM cells. We showed for the first time that ibrutinib induces autophagy in GBM cells both in vitro and in vivo. In addition, we clearly demonstrated that ibrutinib induces autophagic cell death through a process that appears to involve suppression of the Akt/mTOR signaling pathway. The cytotoxicity of ibrutinib was enhanced when autophagy was inhibited by an *Atg7* knock-down or 3MA treatment. Our findings provide important insights into using an anticancer agent in combination with autophagy inhibitor to treat malignant glioma. These observations will aid the development of new chemotherapeutic drugs and designing novel strategies for the treatment of GBM by targeting autophagy in the correct context.

## Additional file

Additional file 1: Figure S1. BTK expression is elevated in GBM patients. (**a**) Relative BTK levels analyzed in GBM specimens vs. normal brain tissues deposited in the Cancer Genome Atlas (TCGA). (**b**) BTK levels in GBM patient tissues deposited in Gene Expression Omnibus (GEO). (TIF 716 kb)

## Abbreviations

3MA: 3-Methyladenine
AMPK: AMP-activated protein kinase
BTK: Bruton’s tyrosine kinase
CA-Akt: Constitutively active Akt
CCK-8: WST-8 cell counting kit-8
CQ: Chloroquine
EdU: 5-Ethynyl-2′-deoxyuridine
GBM: Glioblastoma
GEO: Gene expression omnibus
IHC: Immunohistochemistry
si-Atg7: Small interfering RNA targeting *Atg7*
TCGA: The Cancer Genome Atlas
TEM: Transmission electron microscopy
