# Correction: Ibrutinib, a Bruton’s tyrosine kinase inhibitor, exhibits antitumoral activity and induces autophagy in glioblastoma

**DOI:** 10.1186/s13046-025-03304-y

**Published:** 2025-02-10

**Authors:** Jin Wang, Xiaoyang Liu, Yongzhi Hong, Songtao Wang, Pin Chen, Aihua Gu, Xiaoyuan Guo, Peng Zhao

**Affiliations:** 1 Department of Neurosurgery, The First Affiliated Hospital, Nanjing Medical University, Nanjing, 210000 China; 2 Department of Intensive Care Unit, School of Medicine, Shanghai General Hospital, Shanghai Jiao Tong University, Shanghai, 201620 China; 3 Department of Neurosurgery, Northern Jiangsu People’s Hospital, Yangzhou, 211406 China; 4 State Key Laboratory of Reproductive Medicine, Institute of Toxicology, Nanjing Medical University, Nanjing, 210000 China; 5 Key Laboratory of Modern Toxicology of Ministry of Education, School of Public Health, Nanjing Medical University, Nanjing, 210000 China; 6 Department of Neurosurgery, The Affiliated Zhong Da Hospital of Southeast University, Nanjing, 210009 China; 7 Department of Neurosurgery, Shengze Hospital of Jiangsu Province, Suzhou, 215228 China

**Correction: J Exp Clin Cancer Res 36, 96 (2017)**

10.1186/s13046-017-0549-6

Following the publication of the original article [1], the authors identified errors in the images of Figs. 3 and 6, specifically:

- Figure 3b: The nuclear dye should be labeled as DAPI, not DIPA.
- Figure 3b: The positions of the DAPI images for the DMSO group and the 10 µM group have been swapped, and the merged image for the DMSO group is incorrect.
- Figure 6d: The Ki67 IHC image used for the Ib group is incorrect.

The corrected figures are provided below:

The corrections do not affect the overall results, discussion, or conclusion of the article.

**Incorrect Fig.** 3

**Fig. 3:**
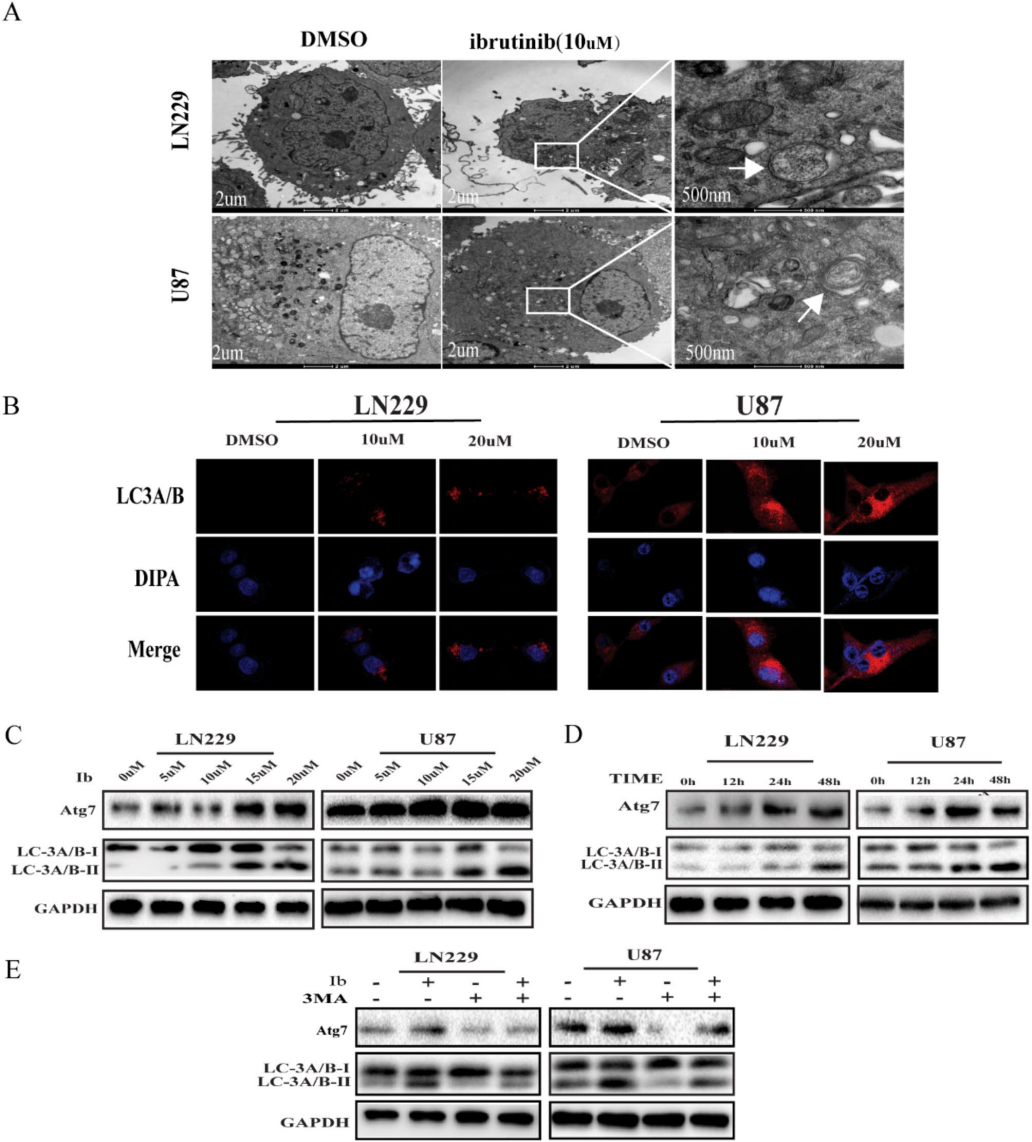
Ibrutinib induces autophagy in GBM cells. (**a**) TEM revealed autophagosome ultrastructures in the enlarged images (arrows) after a 24-h treatment with 10 µM ibrutinib. (**b**) Representative images of immunocytochemistry. Red fluorescence indicates the presence of LC-3 protein. (**c, d**) GBM cells were incubated with different concentrations of ibrutinib for 24 h (**c**) or with 10 µM ibrutinib for various times (**d**), and LC3A/B-II, Atg7, and GAPDH levels were assessed by immunoblotting. (**e**) LC3A/B and Atg7 levels examined by western blot analysis in LN229 and U87 cells after treatment with ibrutinib (10 µM) or DMSO, in the absence or presence of 3MA (2 nM)

**Correct Fig.** 3

**Fig. 3:**
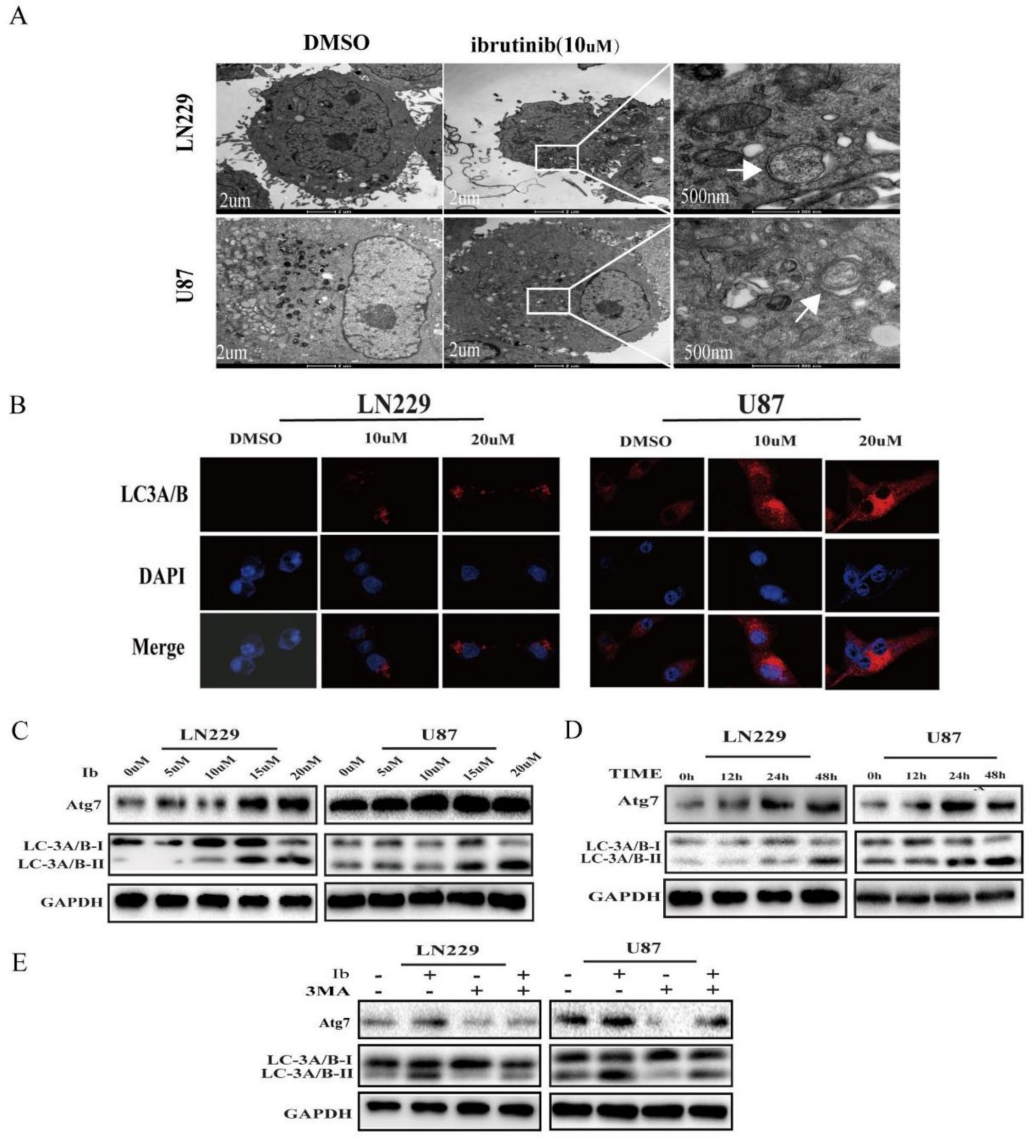
Ibrutinib induces autophagy in GBM cells. (**a**) TEM revealed autophagosome ultrastructures in the enlarged images (arrows) after a 24-h treatment with 10 µM ibrutinib. (**b**) Representative images of immunocytochemistry. Red fluorescence indicates the presence of LC-3 protein. (**c, d**) GBM cells were incubated with different concentrations of ibrutinib for 24 h (**c**) or with 10 µM ibrutinib for various times (**d**), and LC3A/B-II, Atg7, and GAPDH levels were assessed by immunoblotting. (**e**) LC3A/B and Atg7 levels examined by western blot analysis in LN229 and U87 cells after treatment with ibrutinib (10 µM) or DMSO, in the absence or presence of 3MA (2 nM)

**Incorrect Fig.** 6

**Fig. 6:**
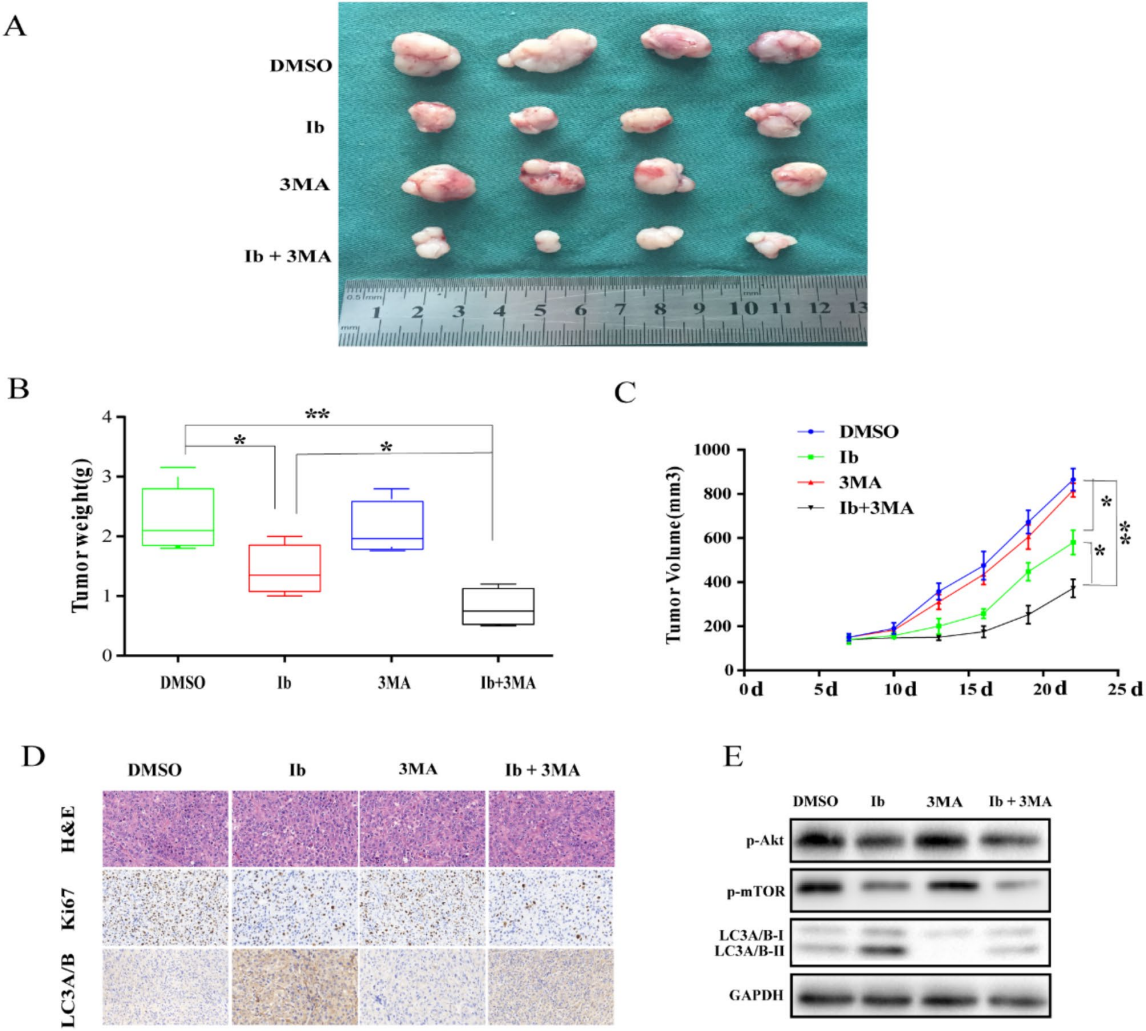
3MA treatment enhances antitumor efficacy of ibrutinib in U87 xenograft model. Mice were sacrificed 22 days after the indicated treatments.The tumors were isolated (**a**), and tumor weight (**c**) and volume (**d**) were measured; *p < 0.05, **p < 0.01. (**e**) Analysis of tumors from each group by H&E staining and immunohistochemical detection of LC3A/B and Ki67. (**f**) Western blot analysis of p-Akt, p-mTOR, mTOR, LC3A/B, and GAPDH levels in isolated tumors

**Correct Fig.** 6

**Fig. 6:**
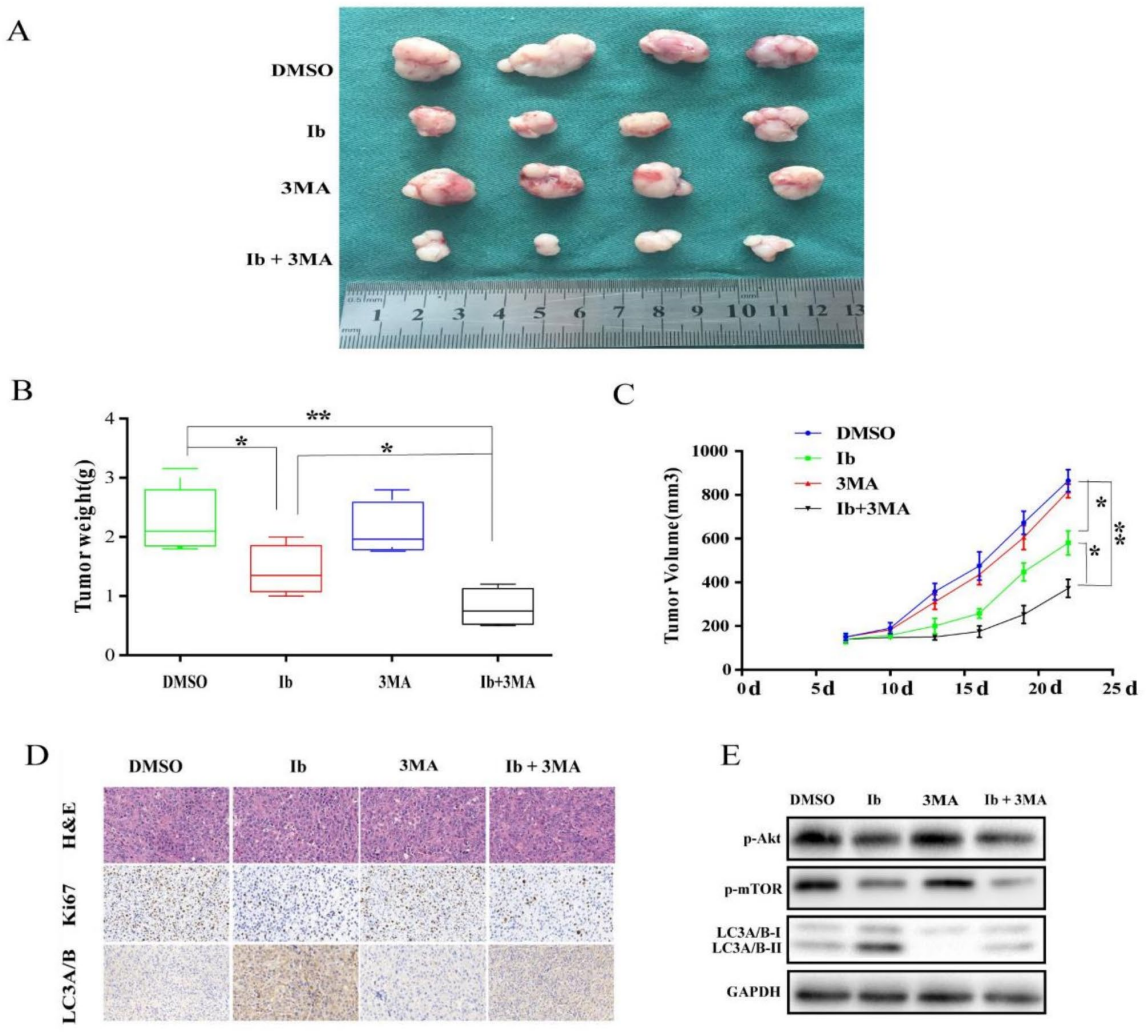
**3**MA treatment enhances antitumor efficacy of ibrutinib in U87 xenograft model. Mice were sacrificed 22 days after the indicated treatments.The tumors were isolated (**a**), and tumor weight (**c**) and volume (**d**) were measured; *p < 0.05, **p < 0.01. (**e**) Analysis of tumors from each group by H&E staining and immunohistochemical detection of LC3A/B and Ki67. (**f**) Western blot analysis of p-Akt, p-mTOR, mTOR, LC3A/B, and GAPDH levels in isolated tumors
