# Supplementary figures and images for: Ibrutinib, a Bruton’s tyrosine kinase inhibitor, exhibits antitumoral activity and induces autophagy in glioblastoma
Source: J Exp Clin Cancer Res. 2017 Jul 17;36:96. doi: 10.1186/s13046-017-0549-6 (PMC5513110; doi:10.1186/s13046-017-0549-6)

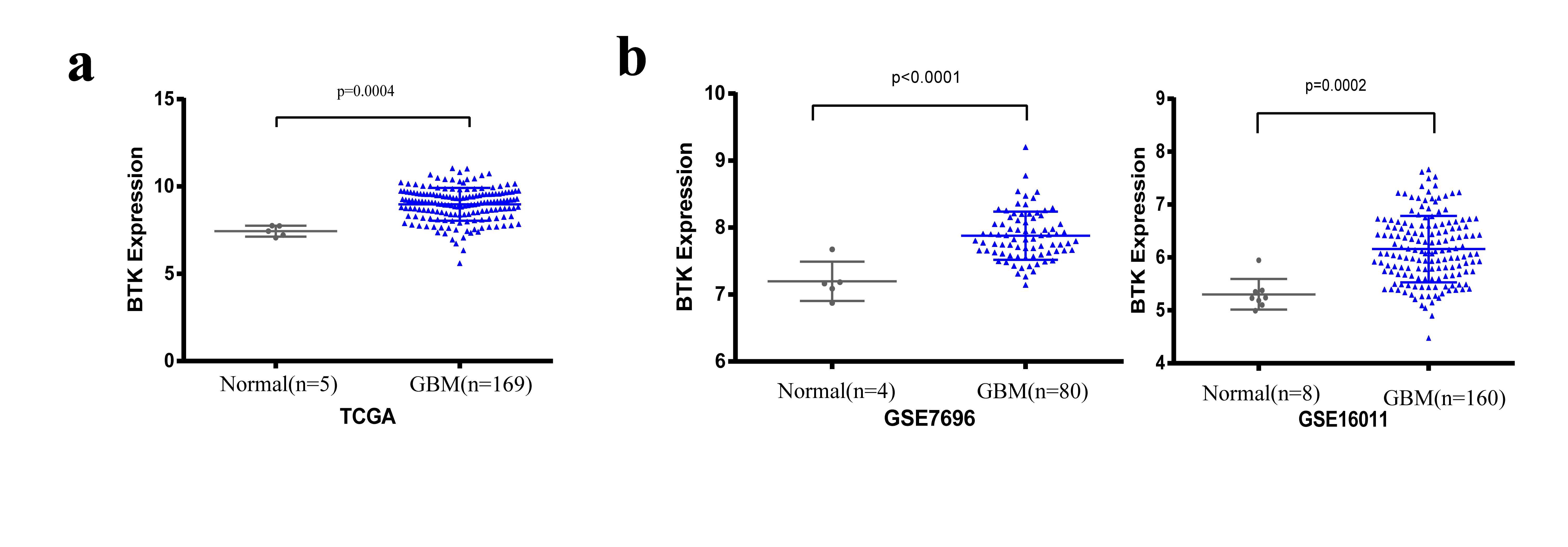

Supplement: Additional file 1: Figure S1. — BTK expression is elevated in GBM patients. (a) Relative BTK levels analyzed in GBM specimens vs. normal brain tissues deposited in the Cancer Genome Atlas (TCGA). (b) BTK levels in GBM patient tissues deposited in Gene Expression Omnibus (GEO). (TIF 716 kb) [file 13046_2017_549_MOESM1_ESM.tif]
